# Health system strategies for adaptation to drought: A global scoping review

**DOI:** 10.1016/j.joclim.2026.100742

**Published:** 2026-09-10

**Authors:** Ahmadreza Khosravifar, Hamidreza Aghababaeian, Ali Bakhtiyari, Mohammad Esmaeil Motlagh, Ali Asgary, Carolyn Stephens, Forozan Bahari Babadi, Fateme Yazdi, Abbas Ostadtaghizadeh

**Affiliations:** a Student Research Committee, Dezful University of Medical Sciences, Dezful, Iran; b Center for Climate Change and Health Research (CCCHR), Dezful University of Medical Sciences, Dezful, Iran; c Department of Health in Emergencies and Disasters, Dezful University of Medical Sciences, Dezful, Iran; d Universal Scientific Education and Research Network (USERN), Dezful, Iran; e Department of Emergency Medicine, School of Medicine, Dezful University of Medical Sciences, Dezful, Iran; f Ahvaz Jundishapour University of Medical Sciences, Ahvaz, Iran; g Disaster and Emergency Management, School of Administrative Studies, York University, Toronto, Canada; h UCL Bartlett Development Planning Unit, London School of Hygiene & Tropical Medicine, London, UK; i Department of Medical Emergencies, School of Nursing, Dezful University of Medical Sciences, Dezful, Iran; j Department of Health in Emergencies and Disasters, Tehran University of Medical Sciences, Tehran, Iran; k Climate Change and Health Research Center (CCHRC), Tehran University of Medical Sciences, Tehran, Iran

**Keywords:** Adaptation, Drought, Climate change, Disasters, Health, Health system

## Abstract

**Introduction:**

Drought poses substantial risks to population health and to the capacity, continuity, and performance of health systems. This scoping review aimed to systematically identify, map, and categorize the available evidence on health-system adaptation strategies for drought and to highlight major gaps in the evidence base.

**Methods:**

Following JBI guidance for scoping reviews and the PRISMA-ScR reporting guideline, Web of Science, PubMed, Scopus, Google Scholar, and selected gray-literature sources were searched without date restrictions up to March 2025. Two reviewers independently screened records and full-text sources against predefined eligibility criteria. Data were charted using a standardized form and analyzed through inductive qualitative content analysis and thematic categorization. Critical appraisal was conducted to describe the methodological characteristics of the included evidence and support cautious interpretation; appraisal findings were not used as exclusion criteria.

**Results:**

Of the 7,334 records identified, 36 evidence sources met the eligibility criteria and were included. The evidence base comprised peer-reviewed studies and reviews, a book chapter, an institutional report, and gray-literature documents. Thematic categorization identified ten domains: assessment and monitoring; mental health and social support; water-resource management; food security and agriculture; health-infrastructure resilience; intersectoral coordination and governance; education and awareness; emergency response and crisis management; innovation and technology; and economic support and policy.

**Conclusion:**

This review provides a structured map of reported health-system adaptation strategies for drought and demonstrates the breadth and multidimensional nature of the available evidence. However, evidence regarding implementation, effectiveness, scalability, and applicability in low-resource settings remains limited.

## 1 Introduction

Drought is a climatic phenomenon characterized by a prolonged period of abnormally dry conditions resulting from sustained reductions in water availability for human and environmental needs [1,2]. It is increasingly recognized not only as an environmental hazard but also as a major public health threat [3]. Climate projections indicate that global warming will intensify the frequency, duration, and severity of drought events through increased evaporation and surface drying, particularly during warmer seasons [2]. Recent assessments by the Intergovernmental Panel on Climate Change (IPCC) confirm with high confidence that drought risk will increase across many regions under all emission scenarios, exacerbating existing vulnerabilities in water, food, and health systems [4].

Drought drives widespread environmental degradation, including reduced water quality, loss of wetlands, soil erosion, ecological habitat destruction, increased wildfire risk, biodiversity loss, and ecological collapse [2,5,6]. These disruptions elevate population-level health risks through both direct and indirect pathways [[7], [8], [9]]. The health consequences of drought are complex, multifactorial, and often long-term. Evidence indicates that drought exacerbates chronic diseases [10], worsens respiratory conditions by increasing airborne particulates and pollutants [5], and elevates the risk of vector-borne, water-borne, and food-borne diseases [2,5]. Nutritional deficiencies and food insecurity associated with prolonged drought have been linked to excess mortality [10]. Between 1970 and 2019, approximately 650,000 deaths were attributed to drought, making it one of the deadliest climate-related hazards globally [11].

Beyond its direct health effects, drought places substantial strain on health systems. Increased hospitalization rates, rising disease burdens, and heightened mental health needs disrupt routine service delivery and intensify workforce pressures. Health facilities may experience operational disruptions due to water shortages, compromised sanitation systems, and unstable energy supply—factors that directly threaten service continuity and patient safety [12]. These impacts are particularly severe in low- and middle-income countries (LMICs), where health systems often operate with limited surge capacity, constrained financing, and pre-existing infrastructure gaps [13]. Water scarcity may further compromise cold chain systems and essential medical equipment, thereby weakening preparedness and response capacity [1].

Globally, the scale of exposure is considerable. In 2022, more than 2.3 billion people faced water stress, including nearly 160 million children exposed to severe and prolonged drought [11]. Between 2000 and 2023, over 1.6 billion people were affected by drought worldwide [14]. Moreover, the World Health Organization estimates that climate change may contribute an additional 250,000 deaths annually between 2030 and 2050, primarily through malnutrition, malaria, diarrhea, and heat stress [15]. These projections underscore the urgent need to strengthen health system resilience in the face of climate-sensitive hazards, particularly slow-onset disasters such as drought [16,17].

According to the Intergovernmental Panel on Climate Change (IPCC), some climate-related impacts are now unavoidable, making proactive adaptation essential across sectors, including health [18]. Adaptation refers to adjustments in systems, processes, and practices to moderate the negative impacts of actual or expected climate conditions or to take advantage of potential benefits [19]. In the health sector, adaptation encompasses measures such as strengthening early warning systems, implementing climate-informed surveillance, protecting water and sanitation infrastructure, safeguarding supply chains, reinforcing workforce capacity, and enhancing governance mechanisms [4,12]. The World Health Organization has emphasized the concept of “climate-resilient health systems,” defined as systems capable of anticipating, responding to, coping with, recovering from, and adapting to climate-related shocks while maintaining core functions and essential service delivery [12]. Despite growing recognition of climate-resilient health systems, the existing literature remains fragmented. Most studies address climate change adaptation broadly, without distinguishing drought-specific health system strategies. Furthermore, adaptation research frequently prioritizes community-level or agricultural interventions, with comparatively limited attention to system-level governance, financing, service delivery, and infrastructure resilience within the health sector [20]. To the best of the authors’ knowledge, no previous scoping review has comprehensively mapped and categorized health system–focused adaptation strategies specifically addressing drought as a distinct climate hazard. This represents both a critical knowledge gap and a policy-relevant blind spot.

Implementation of adaptation measures is often hindered by weak institutional coordination, financial constraints, limited technical capacity, and insufficient data for informed decision-making [21,22]. As drought events intensify and disproportionately affect vulnerable populations—including children, older adults, and rural communities [23,24]—health systems require structured, evidence-informed strategies and drought awareness to strengthen preparedness and adaptive capacity. A consolidated and systematic mapping of the available evidence is therefore essential to inform policy prioritization, strategic investment, and resilience-building across core health system functions. This scoping review aimed to systematically identify, map, and categorize the available evidence on health system adaptation strategies for drought, highlight key gaps in the evidence base, and develop a structured thematic framework to inform policy, planning, and resilience-strengthening efforts. By organizing adaptation strategies across health system domains, this review seeks to provide researchers, policymakers, and health planners with a comprehensive overview of the available evidence relevant to drought-related health system adaptation.

## 2 Methods

This scoping review was conducted in accordance with JBI guidance for scoping reviews [25] and reported according to the PRISMA Extension for Scoping Reviews (PRISMA-ScR) [26]. The original protocol was registered in PROSPERO as a systematic review (CRD42022308953). Following methodological reassessment of the review objective and synthesis approach, the completed review was more appropriately classified and reported as a scoping review. The core research topic, search scope, and objective of identifying health-system adaptation strategies remained unchanged.

A comprehensive search was conducted in Scopus, PubMed, and Web of Science (WoS), as well as Google Scholar and gray literature sources. Gray literature searches focused on publicly available reports, guidelines, frameworks, toolkits, and other relevant documents from international organizations, including World Health Organization (WHO), Food and Agriculture Organization (FAO), United Nations (UN) agencies, and the World Bank [4,11,[27], [28], [29], [30], [31]]. To ensure comprehensive retrieval, both backward (reference list screening) and forward citation searching were performed. Manual searches of key journals and reference lists of relevant reviews and included evidence sources were also conducted to identify sources not indexed in the databases.

A standardized search strategy was developed in collaboration with a specialist librarian using MeSH terms, relevant controlled vocabulary, and free-text terms related to drought, health systems, health, and adaptation, combined with Boolean operators. Search terms were tailored to each database to maximize sensitivity. The full search syntax is provided in Supplementary File 1.

The search had no temporal restrictions, covering all literature up to March 2025. Only English-language documents were included, as the research team lacked capacity to reliably assess non-English full texts. The review question was developed using the Population–Concept–Context framework. The population included health systems, healthcare facilities, public health agencies, communities, and populations affected by drought, provided that the reported strategy had an explicit connection to health-sector functions, public health practice, healthcare delivery, or health-system planning. The concept comprised strategies, measures, and actions intended to support health-system adaptation to drought. The context included healthcare, public health, environmental health, and relevant community settings globally.

The definition of "health system" followed established frameworks and included healthcare providers and facilities, public health services, environmental and sanitation services, health promotion activities, supply chains, long-term care, and financing structures [20,21,32].

### 2.1 Eligibility criteria

#### 2.1.1 Inclusion criteria

Eligible evidence sources included primary quantitative, qualitative, and mixed-methods studies; reviews; case studies; policy and technical reports; organizational guidance documents; and relevant gray literature published up to March 2025, provided that they reported at least one strategy relevant to health-system adaptation to drought.

The primary concept of interest was drought-related health-system adaptation strategies. Additional information of interest included contextual factors influencing adaptation, implementation considerations, and reported health-related impacts.

#### 2.1.2 Exclusion criteria

Editorials, commentaries, dissertations, conference abstracts, non-research letters, and sources not reporting relevant adaptation strategies were excluded. Sources addressing climate change broadly without drought-specific findings, focusing exclusively on immediate emergency response without an adaptation or resilience component, or lacking a clear connection to health-system functions were also excluded.

### 2.2 Selection of relevant evidence sources

**Fig. 1:**
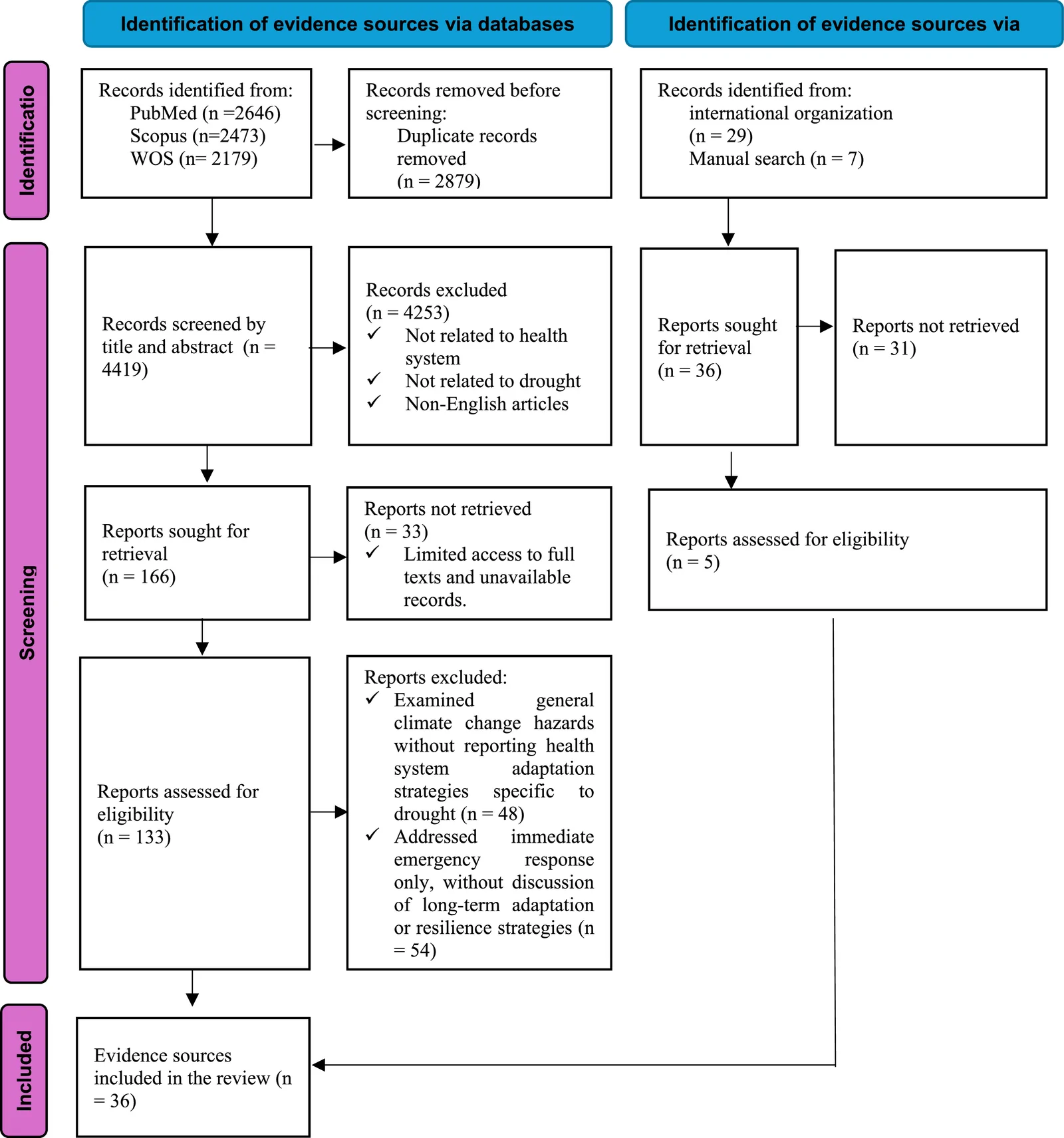
PRISMA-ScR flow diagram of the search and source-selection process.

Duplicates were removed using EndNote software. Two reviewers independently screened titles and abstracts against predefined eligibility criteria, followed by full-text assessment of potentially eligible sources. Any discrepancies were resolved through discussion or consultation with a third reviewer. The source-selection process is summarized in the PRISMA-ScR flow diagram (Fig. 1).

### 2.3 Data charting

A standardized data-charting form was developed by the review team to systematically collect relevant information from the included evidence sources. The form was piloted on a subset of sources and subsequently refined to improve clarity, consistency, and completeness. Charted data included bibliographic characteristics, year of publication, country or geographical setting, source type and study design, population or setting, health-system component, drought-related adaptation strategies, contextual and implementation considerations, and reported health-related outcomes, where available.

Three reviewers (HA, FBB, and AKH) independently charted the data. Any discrepancies were resolved through discussion and consensus or, when necessary, through consultation with a third reviewer. Because the review included heterogeneous evidence sources, the charting form was applied flexibly while maintaining consistency in the core variables extracted across all sources. Evidence sources identified through backward and forward citation searching were documented separately to maintain transparency regarding the route of identification.

### 2.4 Critical appraisal of included evidence sources

The JBI Critical Appraisal Checklists were used by two independent reviewers to assess the methodological characteristics of the included evidence sources. Each source was evaluated using the checklist considered most appropriate to its design, and items were rated as “yes,” “no,” “unclear,” or “not applicable.” Any disagreements between the reviewers were resolved through discussion and, when necessary, consultation with a third reviewer to reach consensus. Appraisal findings were not used as exclusion criteria but were considered when interpreting the evidence. A summary of the appraisal results is presented in Table 1.

**Table 1:** Characteristics of the evidence sources included in the scoping review.

Refs.	Author, Year	Country	Source type/design	Population / Setting	Health System Component	JBI appraisal score
[36]	Mehdipour et al., 2022	Iran	Qualitative case study	57 participants; rural & urban	Public health impacts, environmental health, mental health	7/8
[35]	CDC, EPA, NOAA & AWWA, 2010	USA	Technical guideline / report	Public health agencies; community-level settings	Water management, environmental health, emergency preparedness	6/6
[45]	Sena et al., 2014	Brazil	Narrative review + descriptive analysis	5565 municipalities (1133 semiarid), national public health system	Public health, governance, surveillance, disaster management	6/6
[29]	CDC, 2010	USA	Guide	Public health agencies; community level	Water management, public health preparedness, environmental health	5/6
[28]	WHO, 2019	Global	Checklist / Guide	Health care facilities; health workforce; WASH; energy; infrastructure	Workforce preparedness, WASH systems, energy systems, infrastructure	5/6
[14]	Mani et al., 2024	Global	Retrospective quantitative analysis	Global population; regions (Southern Asia, Sub-Saharan Africa, etc.)	Health system preparedness, emergency response, surveillance	7/10
[54]	Petkova et al., 2017	USA	book chapter	Rural agricultural communities; drought-affected populations	Mental health services; surveillance; community support	5/6
[48]	Wijesekara et al., 2020	Sri Lanka	Cross-sectional survey	90 hospitals	Infrastructure resilience; water & energy systems	9/10
[49]	WHO, 2011	Horn of Africa	Report	Drought-affected populations & displaced communities	Emergency response; surveillance; WASH; disease control; nutrition; vaccination	6/6
[2]	Yusa et al., 2015	Canada	Review	General population	Surveillance; water systems; environmental health; emergency preparedness	5/6
[1]	Grigoletto et al., 2016	Brazil	Descriptive qualitative / documental study	National health sector actions (Brazil)	Health surveillance; water safety; emergency coordination; governance	9/10
[38]	Salvador et al., 2023	SPAIN	Review	General population	Public health; surveillance; environmental health	6/6
[41]	START Center, 2024	Haiti, Ethiopia, Pakistan	Technical report / narrative synthesis	National health systems & vulnerable populations	Public health preparedness; WASH; food security; surveillance	6/10
[39]	Sari et al., 2019	Indonesia	Qualitative case study	3 Public Health Centers (PHCs) in Padang	Primary health services; emergency preparedness; environmental health	7/8
[50]	Lombe et al., 2024	Sub-Saharan Africa	Systematic review	93 studies (2010–2024)	Public health relevance via drought impacts	7/10
[55]	Andriesse, 2021	Swiss	Technical report / Web page	Farmers & agricultural sectors in drought-affected regions	Agriculture health risk, livelihood protection, community resilience	5/6
[57]	Enenkel et al., 2015	AUSTRIA	Cross sectional	Drought-prone agricultural regions; humanitarian aid users	Food security; early warning; environmental monitoring	9/10
[58]	Kogan et al., 2019	USA	Cross sectional	Global agricultural regions	Food security monitoring; early warning	10/10
[61]	Abunyewah et al., 2023	Australia	Qualitative study	Alice Springs communities	Mental health & community resilience	9/10
[62]	Hart et al., 2011	Australia	Cross sectional	Rural NSW communities	Mental health services; community resilience	7/10
[63]	Missouri Dept. of Mental Health, n.d.	USA	GUIDE	Farmers and agricultural families	Mental health support	5/6
[52]	Padhy et al., 2015	India	Narrative review	General population; climate-related mental health impacts	Mental health; public health	6/6
[51]	Hernández-López et al., 2024	Colombia	Cross-sectional quantitative study	538 farmers in Tolima region	Community resilience; public health relevance	8/10
[44]	Orievulu & Iwuji, 2022	South Africa	Qualitative (IDIs, KIIs, document analysis)	PLHIV, government officials, rural communities in uMkhanyakude	Governance; coordination; service delivery	9/10
[47]	Quintana et al., 2025	South Africa	Qualitative (31 in-depth interviews)	Health decision-makers at provincial, municipal & national levels	Governance; infrastructure; communication; coordination	9/10
[37]	Rosen et al., 2021	Zambia	Qualitative (IDIs + FGDs)	Women & communities in drought-affected districts	Sexual & reproductive health; community wellbeing	9/10
[30]	IFRC, n.d.	USA	Guidance document	Communities exposed to drought	Public health preparedness; community resilience	4/6
[46]	Elstow et al., 2024	Global	Scoping Review + Narrative Inventory Analysis	Hospitals and hospital facilities worldwide	Health infrastructure resilience	7/10
[60]	Makvandi et al., 2023	China	Modeling / environmental simulation	Urban built environment	impacts the quality of life directly in developing areas	8/10
[59]	CDC, n.d.	USA	Guidance toolkit / Technical resource	Public health professionals, communities in drought-prone areas	Public health communication, risk communication, community awareness	6/6
[27]	EPA, n.d.	USA	Report	Municipal water utilities, communities, public health stakeholders	Water resource management; environmental health; public health preparedness	6/6
[42]	Patz et al., 2014	USA	Narrative review	Global populations; vulnerable groups	Public health preparedness; disease prevention	5/6
[56]	WHO, 2023	Global	Framework / guidance report	Health systems worldwide	Governance; workforce; infrastructure; surveillance; financing	8/10
[53]	Sartore et al., 2007	Australia	Narrative review / clinical guidance	Rural farming communities; GPs	Mental health services; primary care	5/6
[43]	Jedd et al., 2021	Lebanon, Jordan, Morocco, Tunisia	Qualitative policy analysis / governance review	National drought-management institutions; drought-affected communities	Governance; risk management	8/10
[40]	Smith et al., 2014	Brazil	Cross sectional	Children under five; 807 municipalities in the Brazilian Amazon	Surveillance; environmental health	9/10

### 2.5 Data synthesis

Following completion of data charting, the reported drought-related health-system adaptation strategies were analyzed using an inductive qualitative content analysis and thematic categorization approach informed by the principles of Braun and Clarke’s thematic analysis [33]. The purpose of the analysis was to systematically organize and categorize the range of reported adaptation strategies rather than to assess the comparative effectiveness of individual interventions. One reviewer performed the initial coding of the charted adaptation strategies, and a second reviewer independently reviewed and verified the generated codes. Codes reflecting similar actions or concepts were iteratively grouped into subthemes and subsequently organized into broader thematic domains representing major areas of health-system adaptation to drought. The preliminary coding structure, subthemes, and overarching domains were reviewed and refined through discussion among the review team. Any disagreements were resolved through consensus or, when necessary, consultation with a third reviewer. The findings were presented using descriptive numerical summaries, tabular mapping of the included evidence sources, and a thematic framework of drought-related health-system adaptation strategies. Quantitative pooling was neither planned nor undertaken because the objective of the review was to map and categorize the available evidence, and the included sources were heterogeneous in design, purpose, context, and reporting format. An audit trail documenting data-charting decisions, coding, theme development, and subsequent revisions was maintained to enhance transparency and reproducibility [34].

## 3 Results

A total of 7,334 records were identified across the bibliographic databases and other sources. After duplicate removal and title, abstract, and full-text screening, 36 evidence sources met the eligibility criteria and were included in the final synthesis (Fig. 1). The included sources comprised 25 peer-reviewed journal articles, one book chapter, five institutional reports, and five gray-literature documents, including guidelines, toolkits, and organizational web publications. Reasons for exclusion at the full-text stage included: (i) sources addressing climate change broadly without drought-specific findings on health-system adaptation, (ii) sources limited to immediate disaster response without an adaptation or resilience component, and (iii) sources not directly related to health-system functions. These exclusion categories are presented in the PRISMA-ScR flow diagram. The included evidence sources were published between January 2007 and March 2025, with an increase in publication activity after 2015 and a peak in 2021 (*n* = 6).

### 3.1 Summary of included evidence sources

The characteristics of the included evidence sources are summarized in Table 1. Geographically, the evidence represented multiple regions, including North America, South America, Europe, Sub-Saharan Africa, South Asia, the Middle East and North Africa, and Oceania. The United States accounted for the highest number of country-specific evidence sources. When categorized by World Bank income classification, the majority of country-specific evidence sources originated from high-income countries (HICs), particularly the United States, Australia, Canada, Austria, and parts of Europe. Upper-middle-income countries (e.g., Brazil, China, Lebanon, Jordan, Morocco, Tunisia, Colombia, South Africa) were moderately represented, whereas lower-middle-income and low-income countries (e.g., Ethiopia, Haiti, Zambia, parts of Sub-Saharan Africa and South Asia) were comparatively underrepresented. This distribution indicates a potential imbalance in the evidence base, with adaptation strategies more frequently documented in higher-income settings.

Across the 36 included sources, the most frequently addressed domain was assessment, surveillance, monitoring systems, and preparedness strategies (*n* = 11) [2,14,27,29,[35], [36], [37], [38], [39], [40], [41], [42]]. Six evidence sources addressed health infrastructure resilience, with particular emphasis on water supply systems, energy security, and facility-level preparedness [1,[43], [44], [45], [46], [47]]. Another six sources focused on broader health system vulnerability and resilience, addressing workforce capacity, governance structures, and service delivery continuity [28,[47], [48], [49], [50], [51]]. Five sources explored climate-related mental health impacts and psychosocial consequences, while three specifically addressed the provision of mental health services during drought conditions [14,41,[52], [53], [54]]. Economic support mechanisms and drought-related policy frameworks were discussed in three sources [38,55,56]. Food security and agricultural strategies with direct or indirect health system relevance were addressed in two sources [57,58], as were education and awareness initiatives [30,59]. Only one source primarily emphasized innovation and technological modeling approaches, indicating that technology-driven adaptation remains comparatively underrepresented in the current evidence base [60]. Most included sources used qualitative, narrative, descriptive, or guidance-oriented approaches, whereas a smaller proportion comprised quantitative observational studies, including cross-sectional designs. Critical appraisal indicated variability in methodological characteristics across the included evidence sources, with many sources meeting most of the applicable appraisal criteria.

### 3.2 Thematic synthesis

**Fig. 2:**
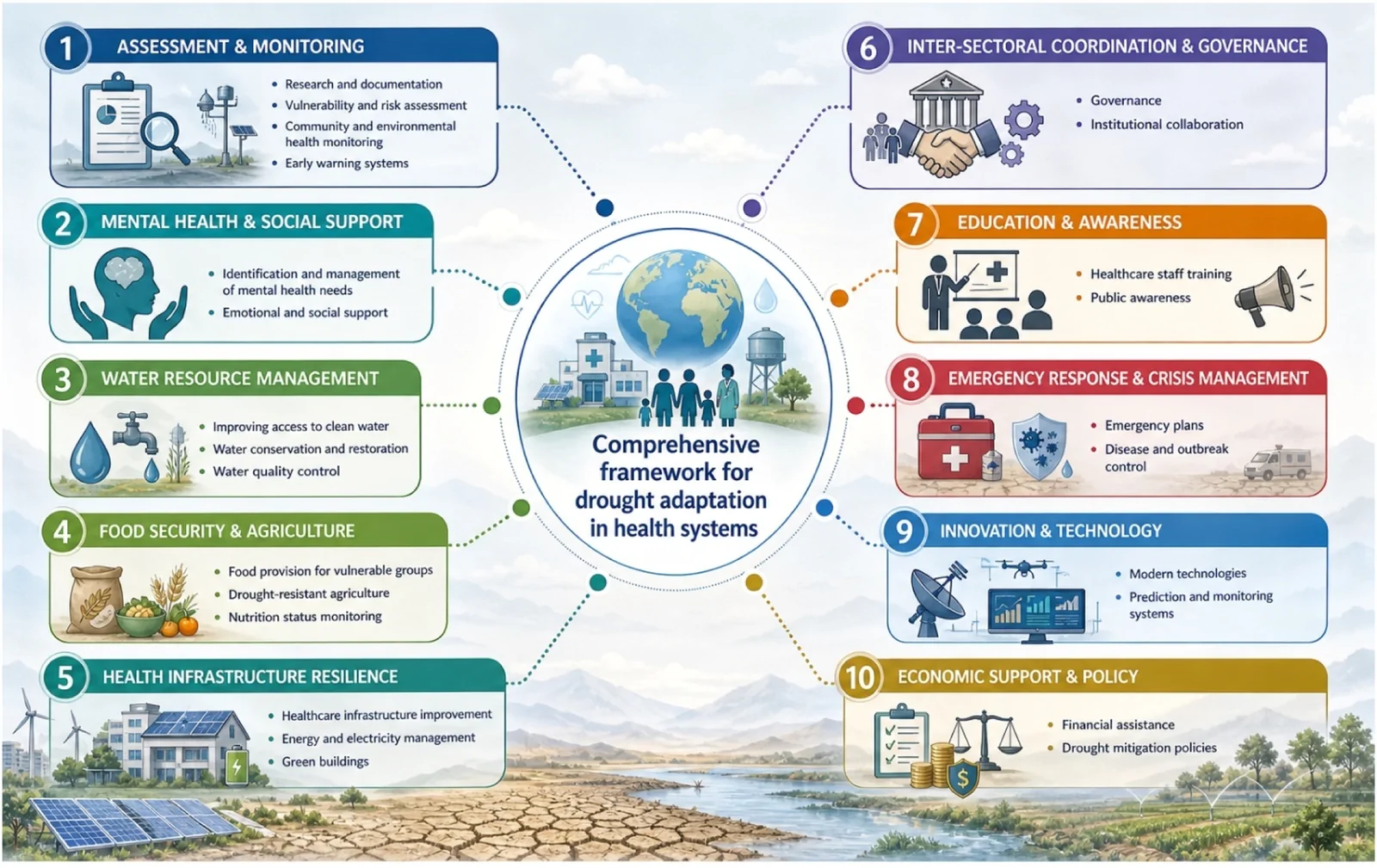
Comprehensive framework for drought adaptation in health systems.

Following systematic data charting and thematic analysis, drought-adaptation strategies were organized into ten overarching domains, each comprising multiple subthemes reflecting key areas of health-system resilience. A comprehensive list of coded actions is provided in Supplementary File 2, and the thematic structure is illustrated in Fig. 2.

In the assessment and monitoring domain, strategies are grouped into research and documentation, vulnerability and risk assessment, community and environmental health monitoring, and early warning systems, covering actions such as evidence generation, risk evaluation, surveillance activities, and development of forecasting systems [2,14,27,29,[35], [36], [37], [38], [39], [40], [41], [42]]. The mental health and social support domain includes identification and management of mental health needs and emotional and social support, encompassing actions related to mental health service provision, community-based interventions, and psychosocial support mechanisms [14,41,[52], [53], [54]]. Within water resource management, subthemes include improving access to clean water, water conservation and restoration, and water quality control, incorporating actions such as water supply provision, conservation measures, and monitoring and management of water safety [1,[43], [44], [45], [46], [47]].

The food security and agriculture domain consists of food provision for vulnerable groups, drought-resistant agriculture, and nutrition status monitoring, including actions related to food assistance, agricultural adaptation, and nutritional surveillance [57,58]. The health infrastructure resilience domain includes healthcare infrastructure improvement, energy and electricity management, and green buildings, covering actions aimed at strengthening facility capacity, ensuring energy continuity, and improving environmental sustainability [43,44,46,47].

In intersectoral coordination and governance, subthemes of governance and institutional collaboration include actions related to policy development, coordination mechanisms, communication systems, and stakeholder engagement [28,[47], [48], [49], [50], [51]]. The education and awareness domain comprises healthcare staff training and public awareness, including actions focused on capacity building, training programs, and dissemination of health-related information [30,59]. The emergency response and crisis management domain includes emergency plans and disease and outbreak control, encompassing actions related to service continuity, emergency response planning, and disease prevention measures [14,49]. In the innovation and technology domain, subthemes include modern technologies and prediction and monitoring systems, covering actions such as technological applications, data-driven systems, and advanced monitoring tools [60]. Finally, the economic support and policy domain includes financial assistance and drought mitigation policies, incorporating actions related to financial support mechanisms, subsidies, and policy-level interventions [38,55,56].

### 3.3 Geographic distribution of included evidence sources

**Fig. 3:**
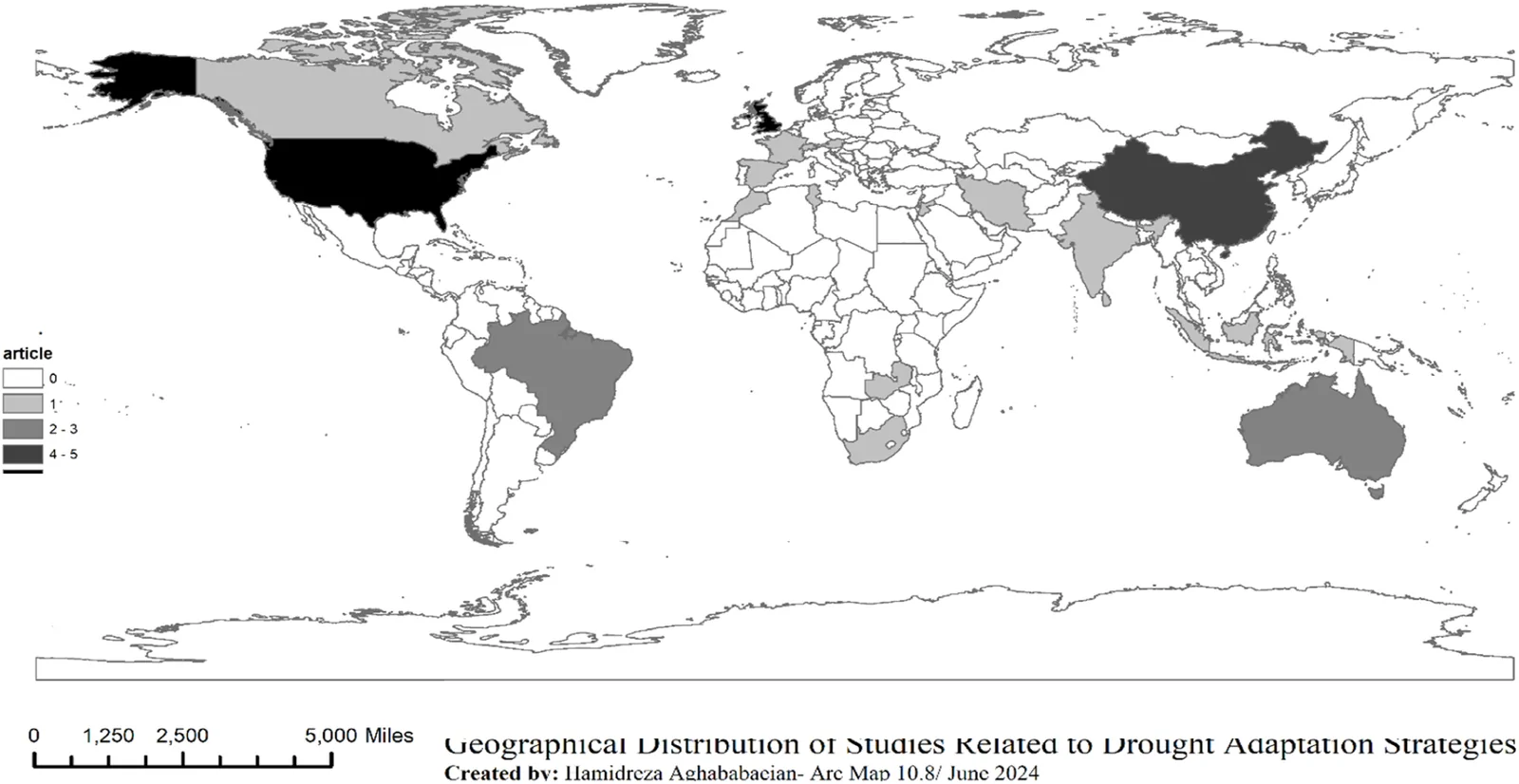
Geographical distribution of included evidence sources on health-system adaptation strategies for drought.

Fig. 3 shows the geographic distribution of the included evidence sources. The United States had the highest number of publications (*n* = 8), followed by Australia and Brazil. Comparative analysis by World Bank income groups revealed that most evidence sources originated from high- and upper-middle-income countries, highlighting potential gaps in low-income regions.

## 4 Discussion

Based on the authors’ knowledge and a systematic search of major scientific databases, this review represents one of the first comprehensive mappings specifically focused on health-system adaptation strategies for drought, addressing a gap in the literature that has largely emphasized broader climate change adaptation rather than drought-specific responses [[64], [65], [66]]. The identification of ten interconnected domains underscores the systemic nature of drought impacts and indicates that reported adaptation approaches extend beyond isolated interventions to encompass coordinated, multilevel responses across health-system components. This review highlights that drought adaptation in health systems is multidimensional and involves core system functions, including surveillance, service delivery, infrastructure, governance, and financing. Rather than being reported as isolated interventions, the identified strategies span multiple system levels, suggesting the importance of integration between technical, organizational, and community-based responses. This systems-oriented perspective extends previous climate–health literature by illustrating how drought-specific risks are addressed across multiple health-system functions.

A notable trend observed was the increase in publications after 2021, likely reflecting growing global recognition of climate-related health risks, improved availability of climate and health data, and heightened awareness of system vulnerabilities following the COVID-19 pandemic.

The predominance of studies from high-income countries—particularly the United States—highlights a persistent imbalance in the evidence base, despite the disproportionate burden of drought in low- and middle-income settings. This imbalance raises important concerns regarding the generalizability of identified strategies, as many interventions—particularly those relying on advanced technologies and infrastructure—may not be directly transferable to low-resource settings where drought impacts are often more severe. This highlights a critical equity gap and underscores the need for context-specific adaptation approaches in low- and middle-income regions. Importantly, this imbalance may also bias the types of adaptation strategies identified, favoring technologically intensive and resource-dependent interventions that may not be feasible in low-resource settings. Across the included evidence sources, limited evidence was found regarding the evaluation of adaptation effectiveness, with most sources focusing on proposed or implemented strategies rather than measured outcomes. Accordingly, the identified domains should be interpreted as a map of reported adaptation strategies rather than as a hierarchy of interventions with established effectiveness. Where reported, barriers to implementation included financial constraints, limited infrastructure, weak intersectoral coordination, and insufficient local capacity, particularly in low- and middle-income settings. These findings indicate that, despite the diversity of identified strategies, their real-world scalability and effectiveness remain insufficiently examined. This limited evidence on effectiveness restricts the ability to prioritize interventions and highlights the need for more implementation-focused and context-sensitive research.

### 4.1 Assessment & monitoring

Assessment and monitoring emerged as a foundational domain through which health systems identify risks, anticipate impacts, and guide resource allocation. Vulnerability assessments, epidemiological investigations, and environmental health monitoring were consistently identified as essential components of this process [28,29,35]. These functions support early detection of changes in disease patterns, water quality, and population health, thereby strengthening the surveillance and intelligence function of health systems and enabling more targeted and timely interventions. The importance of integrated governance frameworks and intersectoral coordination in risk assessment has been emphasized in prior studies [67,68], which is consistent with the role of early warning systems identified in this review [39,49]. Linking climate data with health surveillance systems enhances the capacity for anticipatory action and improves decision-making processes. By embedding drought monitoring within routine health system planning, these strategies contribute to a shift from reactive response toward proactive and risk-informed adaptation.

### 4.2 Mental health & social support

Drought-related stress, anxiety, and reduced coping capacity underscore the importance of incorporating mental health services into health system adaptation strategies. Studies consistently highlight the need for early identification of psychological impacts, provision of financial support for affected populations, and delivery of culturally sensitive mental health interventions [29,52,54,[61], [62], [63]]. These approaches strengthen the service delivery and health workforce functions of health systems by expanding access to care and improving responsiveness to emerging psychosocial needs.

This domain is particularly relevant in rural and agricultural communities, where drought disrupts livelihoods and amplifies social and economic stressors. Strengthening community networks, expanding access to mental health services, and enhancing practitioner training have been identified as key strategies [69,70]. In addition, integrating mental health into broader drought preparedness and response planning can improve system responsiveness and contribute to more comprehensive and inclusive resilience-building efforts.

### 4.3 Water resource management

Water resource management is a critical domain for maintaining healthcare service delivery during drought conditions. Ensuring access to safe and reliable water supplies is fundamental for infection prevention, sanitation, and continuity of care. Strategies identified in the literature—including drilling new wells, rainwater harvesting, improving storage capacity, and emergency water distribution—support the service delivery function of health systems by enabling uninterrupted clinical operations [28,35,37,44]. In addition to these conventional approaches, the findings highlight the emergence of innovative and sustainability-oriented solutions, such as salt-resistant algae cultivation and permeable urban infrastructure [30,31]. These approaches reflect a broader shift toward integrating environmental and technological strategies within health system adaptation efforts.

Furthermore, the alignment of water management policies with public health objectives has been emphasized as a key component of long-term resilience [71,72]. Embedding water resource strategies within health system planning frameworks strengthens system capacity to respond to both immediate and prolonged drought-related challenges.

### 4.4 Food security & agriculture

Food insecurity during drought has significant implications for nutritional status, disease susceptibility, and long-term health outcomes. Within the scope of this review, these strategies were considered relevant when they were explicitly linked to public health, nutritional surveillance, service delivery, or health-sector planning. Health systems play a central role in addressing these impacts through monitoring malnutrition, coordinating food distribution, and supporting community-based nutrition programs. These actions reinforce the service delivery and public health functions of health systems by linking clinical care with population-level interventions.

The findings are consistent with previous studies emphasizing the importance of drought-resistant crops, improved food storage systems, and alignment with emergency nutrition policies [28,37,42]. In addition, maintaining food quality and safety becomes increasingly critical under conditions of disrupted supply chains [73]. Integrating nutritional surveillance with drought monitoring systems enhances early identification of at-risk populations and supports more timely and targeted interventions, thereby strengthening the overall responsiveness of health systems to drought-related health risks.

### 4.5 Health infrastructure resilience

Health infrastructure resilience is essential for maintaining continuity of care under conditions of water scarcity and energy disruption. Key strategies identified in this review include facility assessment systems, maintenance of reliable energy supply, and improved organizational resource management [28,45]. These interventions strengthen the service delivery and system capacity functions of health systems by ensuring that essential services remain operational during periods of environmental stress.

The use of renewable energy systems—beyond general energy reduction approaches—supports the uninterrupted functioning of critical services such as refrigeration, diagnostics, and life-saving equipment. In addition, incorporating green building designs contributes to long-term climate resilience by reducing vulnerability to heat and resource constraints [48]. Overall, strengthening infrastructure resilience reduces system fragility and enhances the ability of health systems to sustain service delivery during prolonged and recurrent drought conditions.

### 4.6 Intersectoral coordination & governance

Effective drought adaptation depends on strong governance mechanisms and coordinated action across sectors, particularly water, agriculture, and public health. The findings reinforce the central role of governance in aligning policies, resources, and institutional responsibilities to support adaptation efforts. Engaging communities and local institutions enhances risk identification, planning processes, and coordinated response, as highlighted in previous studies [45,61,74]. These mechanisms strengthen the governance and leadership function of health systems by improving coordination, accountability, and communication across sectors. In particular, decentralized decision-making and active community participation enable more context-specific and responsive interventions, which are especially critical in resource-limited settings. Strengthening these governance structures enhances the capacity of health systems to implement integrated and sustainable drought adaptation strategies.

### 4.7 Education & awareness

Education and awareness initiatives play a key role in strengthening adaptive capacity by equipping both communities and health professionals with the knowledge and skills needed to respond to drought-related health risks. Public awareness campaigns addressing air quality, nutrition, and water safety, alongside training programs for healthcare workers, support informed decision-making and risk reduction [2,29,49,62]. These efforts contribute to the health workforce and health promotion functions of health systems by enhancing professional competencies and promoting behavioral change at the population level. Integrating climate and drought-related content into medical and public health education further supports long-term institutional capacity building [75,76]. Such approaches enable health systems to move beyond short-term responses and develop sustained preparedness for climate-related health challenges.

### 4.8 Emergency response & crisis management

Emergency response and crisis management capacities play a critical role in determining how effectively health systems respond to intensified drought conditions. Key strategies include emergency water supply planning, communication systems, disease surveillance, and rapid mobilization of resources [1,29,35]. These measures support the emergency preparedness and response function of health systems by enabling timely and coordinated action during acute events. Consistent with WHO guidance, preparedness efforts also emphasize laboratory capacity, availability of outbreak investigation tools, and continuity of immunization and surveillance programs [49,53]. Integrating emergency preparedness with routine health system functions enhances overall resilience and reduces fragmentation in response efforts.

This integration supports a shift from reactive crisis management toward more anticipatory and system-wide preparedness approaches, improving the capacity of health systems to manage drought-related health risks alongside other concurrent hazards.

### 4.9 Innovation and technology

Emerging technologies—including digital health tools, mobile applications, satellite surveillance, and predictive platforms—play an increasingly important role in supporting drought adaptation [36,39,45,54]. These tools enhance the ability of health systems to detect early warning signals, anticipate disease outbreaks, monitor water availability, and optimize resource allocation. From a systems perspective, technological innovations strengthen the health information and surveillance functions by improving data integration, timeliness, and decision-making capacity. In particular, the linkage between meteorological and health data systems enables more coordinated and evidence-based responses. Despite their potential, the implementation of such technologies remains uneven across settings, which may contribute to disparities in adaptive capacity, particularly between high- and low-resource contexts.

### 4.10 Economic support & policy

Financial and policy mechanisms are central to enabling and sustaining drought adaptation efforts. Strategies such as risk financing, low-interest loans, resource forecasting, and strategic budget allocation support the implementation of adaptation measures across health system domains [37]. These approaches strengthen the health financing and governance functions by ensuring that adequate resources are available and aligned with adaptation priorities. Policies that integrate national development strategies with climate adaptation objectives further enhance long-term system preparedness. However, without sustained financial commitment and supportive policy frameworks, adaptation efforts are difficult to operationalize and scale, particularly in low-resource settings where competing priorities and capacity constraints are more pronounced.

In conclusion, this synthesis indicates that drought adaptation in health systems is reported not as a singular intervention but as a multidimensional set of strategies spanning surveillance, service delivery, infrastructure, governance, and financing. The findings suggest that integration across these domains may be important for developing coherent and context-sensitive adaptation approaches. As climate variability intensifies, health systems may need to move beyond reactive responses toward anticipatory, data-informed, and equity-oriented adaptation pathways. Bridging current evidence gaps—particularly in low- and middle-income settings—and strengthening the implementation and evaluation of adaptation strategies will be important for informing resilient and responsive health systems under increasing drought pressures. However, because most included sources did not evaluate effectiveness, these findings should be interpreted as a map of reported strategies rather than evidence of the comparative effectiveness of individual interventions.

### 4.11 Research limitations

This review has several limitations. First, the search was restricted to English-language sources, which may have resulted in the omission of relevant evidence from non-English-speaking regions experiencing severe drought conditions. This language restriction, together with the predominance of evidence from high-income countries, may have contributed to the underrepresentation of low- and middle-income settings and may limit the global applicability of the findings. These limitations highlight the need for more inclusive and geographically diverse research. Second, the included evidence sources exhibited considerable methodological diversity in terms of study design, purpose, reporting quality, and outcome definitions. This heterogeneity may have influenced the categorization and synthesis of the identified domains and limited direct comparability across sources. Quantitative pooling was not an objective of this scoping review. Third, many included sources described proposed or implemented adaptation strategies without formally evaluating their implementation or effectiveness. Therefore, the review cannot determine the comparative effectiveness of the identified strategies. Finally, although a comprehensive search strategy was used, some gray literature and non-indexed sources may not have been identified, and publication and availability biases cannot be ruled out. Consequently, certain locally implemented, unpublished, or less accessible adaptation strategies may have been underrepresented in the review.

### 4.12 Future directions

Future research should focus on developing standardized but adaptable frameworks for health-system adaptation to drought across diverse regional contexts. Because health-system structures, resource availability, and climatic vulnerability vary across countries, context-specific studies are needed to determine the feasibility, acceptability, and sustainability of adaptation strategies. Further research should examine barriers and facilitators to implementation in real-world health-system settings, including financial, organizational, technical, and policy-related factors. Evaluating the implementation and effectiveness of existing strategies—such as early-warning systems, mental health services, water-management measures, and infrastructure improvements—also will be important for informing decision-making. Additional research is particularly needed in low- and middle-income countries, where drought impacts may be substantial but the available evidence remains limited. Strengthening local data systems and supporting community-engaged adaptation research may help address these evidence gaps and promote more equitable health-system resilience.

### 4.13 Research limitations

This scoping review provides a comprehensive mapping of health-system adaptation strategies for drought, identifying ten interconnected domains that collectively reflect the multidimensional nature of system resilience. Rather than functioning as isolated interventions, the identified strategies highlight the importance of integrated, system-wide approaches linking monitoring, service delivery, infrastructure, governance, and financing. The findings suggest that embedding these components within routine health-system planning may support continuity of care, protection of vulnerable populations, and timely responses to emerging risks. Importantly, the geographical imbalance in the evidence base underscores the need for context-specific adaptation strategies, particularly in low- and middle-income settings where vulnerability and capacity constraints are substantial. Strengthening the implementation, evaluation, and contextual adaptation of these strategies will be important for advancing resilient, equitable, and responsive health systems under increasing drought pressures.

## Ethical approval

The current study was approved by the Ethics Committee of Dezful University of Medical Sciences (DUMS) (Ethics Code: IR.DUMS.SPH.REC.1401.067), and all methods were performed in accordance with relevant guidelines and regulations.

## Consent for publication

Not applicable.

## Use of AI-assisted language editing tools

During the revision process, the authors used AI-assisted language editing tools, including DeepSeek and ChatGPT, solely for improving language clarity, grammar, and academic expression. All AI-assisted suggestions were carefully reviewed, verified, and revised by the research team, and the authors take full responsibility for the final content, interpretation, and scientific accuracy of the manuscript. Additionally, the AI tool ChatGPT was utilized during the visualization stage and for optimizing the visual quality of Fig. 2.

## CRediT authorship contribution statement

**Ahmadreza Khosravifar:** Writing – review & editing, Writing – original draft, Visualization, Validation, Software, Resources, Methodology, Investigation, Funding acquisition, Formal analysis, Data curation. **Hamidreza Aghababaeian:** Writing – review & editing, Writing – original draft, Supervision, Project administration, Formal analysis, Conceptualization. **Ali Bakhtiyari:** Supervision. **Mohammad Esmaeil Motlagh:** Visualization, Supervision. **Ali Asgary:** Methodology, Formal analysis, Data curation. **Carolyn Stephens:** Validation, Supervision, Conceptualization. **Forozan Bahari Babadi:** Writing – review & editing, Writing – original draft, Methodology, Formal analysis, Data curation. **Fateme Yazdi:** Writing – review & editing, Writing – original draft. **Abbas Ostadtaghizadeh:** Visualization, Validation, Supervision, Project administration, Conceptualization.

## Declaration of competing interest

The authors declare that they have no known competing financial interests or personal relationships that could have appeared to influence the work reported in this paper.

## Data Availability

Not applicable.
