# Supplementary material for: Health system strategies for adaptation to drought: A global scoping review
Source: J Clim Chang Health. 2026 Sep 10;31:100742. doi: 10.1016/j.joclim.2026.100742 (PMC13583712; doi:10.1016/j.joclim.2026.100742)
Supplement: Supplementary file 1 [file mmc1.docx]

**Comprehensive Database Search Strategy and Full Electronic Search Syntax for the Systematic Review**

PUBMED

Search: **(((Drought [Title/Abstract] OR arid*[Title] OR dry spell[Title])) AND ((Adapt*[Title/Abstract] OR Resilien*[Title/Abstract] OR Cop* [Title/Abstract] OR Compatibl* [Title] OR Acclimat*[Title] OR Accommodat*[Title] OR Adjust*[Title] OR Conform*[Title] OR Transform*[Title]))) AND (Method [Title] OR procedure [Title/Abstract] OR approach[Title/Abstract] OR technique [Title/Abstract] OR Practice[Title/Abstract] OR Technique [Title/Abstract] OR way [Title/Abstract] OR manner[Title/Abstract] OR Indicator*[Title/Abstract] OR Component*[Title/Abstract] OR Criteri* [Title/Abstract] OR Determinant*[Title/Abstract] OR Factor* [Title/Abstract])**
("Drought"[Title/Abstract] OR "arid*"[Title] OR "dry spell"[Title]) AND ("adapt*" [Title/Abstract] OR "resilien*"[Title/Abstract] OR "cop"[Title/Abstract] OR "compatibl*" [Title] OR "acclimat*"[Title] OR "accommodat*"[Title] OR "adjust*"[Title] OR "conform*" [Title] OR "transform*"[Title]) AND ("Method"[Title] OR "procedure"[Title/Abstract] OR "approach"[Title/Abstract] OR "Technique"[Title/Abstract] OR "Practice"[Title/Abstract] OR "Technique"[Title/Abstract] OR "way"[Title/Abstract] OR "manner"[Title/Abstract] OR "indicator*"[Title/Abstract] OR "component*"[Title/Abstract] OR "criteri*"[Title/Abstract] OR "determinant*"[Title/Abstract] OR "factor*"[Title/Abstract])

N=2560

WOS

Search: **TI=(Drought) AND TS= (Adapt OR Resilient OR Cop OR Compatible OR Acclimated OR Accommodation OR Adjust OR confirm OR Transform) AND TS=(Method OR procedure OR approach OR technique OR Practice OR Technique OR way OR manner OR Indicator OR Component OR Criteri OR Determinat OR Factor)**

Timespan: All years. Indexes: SCI-EXPANDED, SSCI, A&HCI, ESCI.

N=2183

SCOPUS

**( TITLE-ABS-KEY ( drought ) OR TITLE ( arid ) OR TITLE ( dryspell ) AND TITLEABS-KEY ( adapt ) OR TITLE-ABS-KEY ( resilien ) OR TITLE-ABS-KEY ( cop ) OR TITLE-ABS-KEY ( compatibl ) OR TITLE-ABS-KEY ( acclimat ) OR TITLE-ABS-KEY ( accommodat ) OR TITLE-ABS-KEY ( adjust ) OR TITLE-ABS-KEY ( conform ) OR TITLE-ABS-KEY ( transform ) AND TITLE-ABS-KEY ( method ) OR TITLE-ABS-KEY ( procedure ) OR TITLE-ABS-KEY ( approach ) OR TITLE-ABS-KEY ( technique ) OR TITLE-ABS-KEY ( practice ) OR TITLE-ABS-KEY ( way ) OR TITLE-ABS-KEY ( manner ) OR TITLE-ABS-KEY ( indicator ) OR TITLE-ABS-KEY ( component ) OR TITLE-ABS-KEY ( criteri ) OR TITLE-ABS-KEY ( determinate ) OR TITLE-ABS-KEY ( factor ) )**

(Adapt* OR Resili* OR Cop OR Compatible OR Acclimated TOR Accommodation OR Adjust* OR confirm OR Transform)

AND

(Strateg* OR Action OR Method OR procedure OR approach OR technique OR Practice OR Technique OR way OR manner OR Indicator OR Component OR Criteri OR Determinat OR Factor)

AND

(Health sector OR Health services OR Health*)

N=2481
