# Supplementary material for: Health system strategies for adaptation to drought: A global scoping review
Source: J Clim Chang Health. 2026 Sep 10;31:100742. doi: 10.1016/j.joclim.2026.100742 (PMC13583712; doi:10.1016/j.joclim.2026.100742)
Supplement: Supplementary file 2 [file mmc2.docx]

**Adaptation strategies of the health system to drought**

| Assessment & Monitoring | Research and documentation | \| 1 \| Research development on the effects of drought and its reduction methods on each dimension of health (1) |
| --- | --- | --- |
|  |  | \| 2 \| Documentation and evaluation of all previous responses to drought (2) |
|  |  | \| 3 \| Continuous assessment of responses to determine future actions (3) |
|  | Vulnerability and risk assessment | \| 4 \| Active identification of families or groups that are unable to cope with the effects of drought on their own (3) |
|  |  | \| 5 \| Risk assessments of healthcare centers against drought (2, 4) |
|  |  | \| 6 \| Evaluation of the performance and vulnerability of critical parts of the facility (5) |
|  |  | \| 7 \| Risk assessment of the supply chain of essential medical and non-medical products (5) |
|  |  | \| 8 \| Vulnerability assessment and identification of vulnerable populations (2, 4) |
|  |  | \| 15 \| Assessment of domestic capacity to identify resource requirements for drought-related public health measures (2) |
|  |  | \| 13 \| A comprehensive assessment of the effects of drought and the level of resilience against it (3) |
|  |  | \| 51 \| Create an updated plan to assess water and wastewater infrastructure risks (5) |
|  |  | \| 107\| Conducting long-term health data monitoring (2, 4) |
|  |  | \| 19 \| Enhancing the comprehension of resilience among all community members and stakeholders in the region (4) |
|  |  | \| 79 \| Defining and outlining adaptive capacity for various scenarios (4) |
|  |  | \| 81 \| Collaborating with a climatologist for drought insights (4) |
|  |  | \| 127\| Community-based adaptation strategies and international support (6) |
|  | Community and environmental health monitoring | \| 9 \| Quantification of the effects of drought on the prevalence of mental illness (7) |
|  |  | \| 10 \| Drawing the epidemiological profile of the community to identify adverse changes (3) |
|  |  | \| 11 \| Community assessment for public health emergency response (4) |
|  |  | \| 12 \| Evaluation of public health perceptions and estimation of new needs (4) |
|  |  | \| 44 \| Plan and strengthen the integration of structural and operational safety with green initiatives (8) |
|  |  | \| 99 \| Enhancing epidemiological and health surveillance efforts (3) |
|  |  | \| 100\| Ensuring prompt review of reports on epidemic-prone diseases (9) |
|  |  | \| 108\| Regular monitoring of air particles and issuing air quality alerts (2, 10) |
|  |  | \| 110\| Tracking complications and fatalities from drought impacts (3) |
|  |  | \| 162\| Monitoring drinking water quality and prioritizing vulnerable areas (11) |
|  |  | \| 163\| Monitoring cyanobacteria levels in surface water sources (11) |
|  |  | \| 52 \| Establish a monitoring program for healthcare and treatment centers (5) |
|  |  | \| 105\| Monitoring Forest fires (12) |
|  |  | \| 112\| Providing information on water pollution and air quality (5) |
|  | Early warning systems | \| 98 \| Establishing an early warning system for drought risk assessment (6, 13-15) |
|  |  | \| 101\| Developing an agricultural drought monitoring system (16, 17) |
|  |  | \| 104\| Utilizing next-generation satellites for drought detection (18) |
|  |  | \| 113\| Utilizing advanced data to predict famine and food insecurity (13) |
|  |  | \| 114\| Engaging organizations in creating effective warning systems (13) |
|  |  | \| 120\| Investing in early warning systems based on climate models (3) |
|  |  | \| 103\| Establishing an information exchange system with meteorological services (5) |
|  |  | \| 109\| Ensuring proper implementation of the warning system (3) |
| Mental Health & Social Support | Identification and management of mental health needs | \| 9 \| Quantification of the effects of drought on the prevalence of mental illness (7) |
|  |  | \| 16 \| Identifying the needs to enhance the psychological resilience of communities (19) |
|  |  | \| 17 \| Identifying emerging mental health needs within rural families (20) |
|  |  | \| 18 \| Developing and promoting programs to address youth mental health issues (20) |
|  |  | \| 20 \| Creating and promoting programs to support women affected by drought/climate change-related challenges (20) |
|  |  | \| 21 \| Creating and promoting programs that provide youth with information and activities to address mental health issues (20) |
|  |  | \| 26 \| Encouraging greater involvement of general practitioners in enhancing networks and services related to mental health (20) |
|  |  | \| 27 \| Guaranteeing optimal monitoring of mental health and provision of services in rural communities (7) |
|  |  | \| 28 \| Fostering clinical leadership in developing informational programs related to mental health (20) |
|  |  | \| 29 \| Creating and implementing programs and methods for monitoring and treating mental health issues (20) |
|  |  | \| 30 \| Focusing on educating and promoting community awareness of early mental health interventions (20) |
|  |  | \| 31 \| Encouraging individuals to share their concerns with family, friends, and neighbors (21) |
|  |  | \| 32 \| Sharing personal experiences of psychological conditions in groups to help destigmatize mental health issues (20) |
|  |  | \| 33 \| Enhancing early intervention strategies and pathways for mental health, substance abuse, and alcohol services (20) |
|  |  | \| 34 \| Offering subsidies and guaranteed income during drought seasons to reduce economic and psychological stress on farmers (22) |
|  |  | \| 43 \| Reducing socio-economic vulnerability through financial facilities and agricultural insurance (23) |
|  | Emotional and social support | \| 22 \| Cultivating personal relationships (21) |
|  |  | \| 23 \| Establishing and nurturing important rituals such as mealtimes and holiday celebrations(21) |
|  |  | \| 24 \| Acknowledging the emotions and expressions of the community and workers affected by drought (21) |
|  |  | \| 25 \| Creating and promoting spiritual activities as a source of comfort and support during challenging times (21) |
|  |  | \| 31 \| Encouraging individuals to share their concerns with family, friends, and neighbors (21) |
|  |  | \| 32 \| Sharing personal experiences of psychological conditions in groups to help destigmatize mental health issues (20) |
| Water Resource Management | Improving access to clean water | \| 167\| Installing boreholes and ensuring water truck supply in communities (24) |
|  |  | \| 173\| Ensuring adequate access to drinking water for healthcare staff and patients (5) |
|  |  | \| 176\| Facilitating individual access to water resources (24) |
|  |  | \| 177\| Expanding availability of water wells for consumption (25, 26) |
|  |  | \| 178\| Identifying alternative safe water sources by healthcare facilities (5) |
|  |  | \| 185\| Ensuring uninterrupted supply of safe drinking water (9) |
|  |  | \| 158\| Providing solutions for accessing clean water (2) |
|  |  | \| 164\| Installing on-site water purification systems in healthcare facilities (5) |
|  | Water conservation and restoration | \| 50 \| Establish protocols for the safe supply, transfer, and storage of water resources (5) |
|  |  | \| 53 \| Develop strategies for water conservation and management (5) |
|  |  | \| 54 \| Formulate intra- and inter-sectoral water management plans (5) |
|  |  | \| 55 \| Create an emergency plan for the distribution of safe water (5) |
|  |  | \| 165\| Implementing water conservation and management programs (5) |
|  |  | \| 169\| Implementing strategies to enhance rainwater infiltration (27) |
|  |  | \| 170\| Transitioning to drip irrigation systems (27) |
|  |  | \| 171\| Creating green corridors and using water-permeable asphalt (27) |
|  |  | \| 172\| Implementing rainwater catchment systems at healthcare centers (5, 25, 28) |
|  |  | \| 174\| Covering wells to minimize evaporation (27) |
|  |  | \| 175\| Offering requirements or incentives for water conservation (5) |
|  |  | \| 180\| Strengthening water management systems and improving irrigation techniques (6, 23, 29) |
|  |  | \| 168\| Understanding and protecting water resources (30) |
|  |  | \| 179\| Using alternative resources for high-consumption processes (25) |
|  | Water quality control | \| 159\| Assessing water quality from private sources (4) |
|  |  | \| 160\| Conducting sanitary inspections of water supply sources (11) |
|  |  | \| 161\| Developing plans for securing alternative water sources (2) |
|  |  | \| 166\| Collaborating with well maintenance personnel for water testing (2) |
|  |  | \| 181\| Establishing protocols for wastewater facilities (4) |
|  |  | \| 182\| Innovating low-cost technologies in wastewater recycling (31) |
|  |  | \| 67 \| Establishing a water safety plan for addressing water contamination (5) |
| Food Security & Agriculture | Food provision for vulnerable groups | \| 38 \| Offering food assistance to more vulnerable families (6, 26, 29) |
|  |  | \| 39 \| Ensuring access to essential food resources through multiple contracts with suppliers (5) |
|  |  | \| 40 \| Planning to store and distribute basic food rations (27) |
|  | Drought-resistant agriculture | \| 35 \| Cultivating genetically modified crops resistant to heat and drought (32) |
|  |  | \| 36 \| Identifying and growing early and drought-resistant crops (26) |
|  |  | \| 37 \| Promoting agricultural diversity, including the use of hybrid seeds (6, 23, 26, 29, 33) |
|  |  | \| 115\| Providing seed capital for entrepreneurial initiatives (26) |
|  |  | \| 116\| Providing subsidies for fertilizers and agricultural inputs (26) |
|  |  | \| 180\| Strengthening water management systems and improving irrigation techniques (6, 23, 29) |
|  |  | \| 93 \| Enhancing farmers' access to services (34) |
|  | Nutrition status monitoring | \| 41 \| Continuously monitoring the nutritional status of the population (9, 13) |
|  |  | \| 42 \| Promoting and supporting exclusive breastfeeding for infants (9) |
|  |  | \| 148\| Providing health education on food storage and handling (9) |
| Health Infrastructure Resilience | Healthcare infrastructure improvement | \| 44 \| Plan and strengthen the integration of structural and operational safety with green initiatives (8) |
|  |  | \| 45 \| Investing in green buildings with solar panels and water recycling (25) |
|  |  | \| 46 \| Schedule regular inspections of emergency backup generators (5) |
|  |  | \| 47 \| Develop plans for relocating and deploying supplies during outbreaks (5) |
|  |  | \| 48 \| Organize outdoor work during cooler hours of the day (5) |
|  |  | \| 49 \| Implement an anti-mosquito breeding action plan (5) |
|  |  | \| 56 \| Prepare emergency plans for power outages at healthcare centers (5) |
|  |  | \| 57 \| Design programs to reduce overall energy consumption in healthcare facilities (5) |
|  |  | \| 58 \| Establish contingency plans for lighting and communication systems (5) |
|  |  | \| 59 \| Arrange for patient evacuation to cooling stations (5) |
|  |  | \| 60 \| Implement a program to insulate walls and ceilings of healthcare centers (5) |
|  |  | \| 83 \| Enhancing health centers and training health workers (13) |
|  |  | \| 85 \| Installing emergency backup generators at healthcare facilities (5) |
|  |  | \| 86 \| Using in refrigerators to ensure safety of stored supplies (5) |
|  |  | \| 87 \| Ensuring continuous lighting at healthcare centers during outages (5) |
|  |  | \| 88 \| Implementing reflective ceilings to mitigate heat impacts (5) |
|  |  | \| 89 \| Designing resilient pavements and roofs (5) |
|  |  | \| 94 \| Developing a healthcare facility assessment system (5) |
|  |  | \| 95 \| Establishing protocols for water supply at healthcare facilities (5) |
|  |  | \| 96 \| Compiling heat risk management guidelines (5) |
|  |  | \| 14 \| Development of an easy and fast screening system related to health (9) |
|  |  | \| 69 \| Facilitating immediate consultations and access to consultants (34) |
|  |  | \| 84 \| Enhancing local health system capacity (3) |
|  | Energy and electricity management | \| 56 \| Prepare emergency plans for power outages at healthcare centers (5) |
|  |  | \| 57 \| Design programs to reduce overall energy consumption in healthcare facilities (5) |
|  |  | \| 58 \| Establish contingency plans for lighting and communication systems (5) |
|  |  | \| 87 \| Ensuring continuous lighting at healthcare centers during outages (5) |
|  |  | \| 121\| Investing in renewable energy sources (8, 26) |
|  | Green buildings | \| 44 \| Plan and strengthen the integration of structural and operational safety with green initiatives (8) |
|  |  | \| 45 \| Investing in green buildings with solar panels and water recycling (25) |
|  |  | \| 46 \| Schedule regular inspections of emergency backup generators (5) |
|  |  | \| 47 \| Develop plans for relocating and deploying supplies during outbreaks (5) |
|  |  | \| 48 \| Organize outdoor work during cooler hours of the day (5) |
|  |  | \| 49 \| Implement an anti-mosquito breeding action plan (5) |
|  |  | \| 56 \| Prepare emergency plans for power outages at healthcare centers (5) |
|  |  | \| 57 \| Design programs to reduce overall energy consumption in healthcare facilities (5) |
|  |  | \| 58 \| Establish contingency plans for lighting and communication systems (5) |
|  |  | \| 59 \| Arrange for patient evacuation to cooling stations (5) |
|  |  | \| 60 \| Implement a program to insulate walls and ceilings of healthcare centers (5) |
|  |  | \| 85 \| Installing emergency backup generators at healthcare facilities (5) |
|  |  | \| 86 \| Using thermometers in refrigerators to ensure safety of stored supplies (5) |
|  |  | \| 87 \| Ensuring continuous lighting at healthcare centers during outages (5) |
|  |  | \| 88 \| Implementing reflective ceilings to mitigate heat impacts (5) |
|  |  | \| 89 \| Designing resilient pavements and roofs (5) |
| Inter-sectoral Coordination & Governance | Governance | \| 62 \| Create a health response plan and share it with relevant partners (2) |
|  |  | \| 63 \| Ensuring the involvement of public health in incident management systems (2) |
|  |  | \| 64 \| Engaging the healthcare workforce in strategies to address drought (5) |
|  |  | \| 65 \| Ensuring seamless information flow between the healthcare workforce and policymakers (5) |
|  |  | \| 70 \| Developing a local contingency plan for public health emergencies (11) |
|  |  | \| 73 \| Incorporating the public health sector into climate change planning (12) |
|  |  | \| 74 \| Establishing access to resources for assistance requests (2) |
|  |  | \| 75 \| Establishing resilience in health systems during water scarcity (28) |
|  |  | \| 117\| Allocating resources for drought response objectives (2) |
|  |  | \| 118\| Allocating active human and financial resources (3) |
|  |  | \| 119\| Establishing a sustainable budget for emergency preparedness (5) |
|  |  | \| 61 \| Develop an effective risk communication program (5) |
|  |  | \| 68 \| Creating an effective communication plan for drought-related messages (4) |
|  |  | \| 72 \| Implementing communication channels among managers and the public (11) |
|  |  | \| 111\| Disseminating media information on drought conditions (4) |
|  |  | \| 122\| Collaborating with community leaders for awareness (3) |
|  |  | \| 123\| Ensuring ongoing community engagement in information exchange (3) |
|  |  | \| 124\| Facilitating community collaboration for adaptation strategies (19) |
|  |  | \| 125\| Working with communities to reduce health risks (3) |
|  |  | \| 126\| Identifying local resources available (3) |
|  |  | \| 139\| Sharing drought strategies and recommendations (2) |
|  | Institutional collaboration | \| 183\| Identifying key partners and stakeholders (2) |
|  |  | \| 184\| Identifying key laboratories for infectious disease threats (9) |
|  |  | \| 185\| Ensuring uninterrupted supply of safe drinking water (9) |
|  |  | \| 186\| Coordinating drought response with key partners (2) |
|  |  | \| 187\| Collaborating with local stakeholders to reduce vulnerabilities (3, 25) |
|  |  | \| 188\| Collaborating with agencies for conservation measures during drought (4) |
|  |  | \| 189\| Cooperating with municipalities to promote stormwater absorption (31) |
|  |  | \| 190\| Working with community partners for guidance on water utilities (2) |
|  |  | \| 191\| Coordinating health centers with water supply organizations (5) |
|  |  | \| 192\| Coordinating healthcare centers with energy agencies (5) |
|  |  | \| 193\| Early engagement with agencies about public health activities (4) |
|  |  | \| 194\| Promoting health sector involvement in water resource infrastructure (3) |
|  |  | \| 195\| Encouraging organizations to include public health in drought responses (4) |
|  |  | \| 196\| Establishing partnerships for coordinated action (3) |
|  |  | \| 197\| Joining regional drought planning task forces (4) |
|  |  | \| 198\| Establishing communication channels between health professionals and managers (11) |
|  |  | \| 199\| Participating in risk assessment and scenario planning (3) |
|  |  | \| 200\| Actively participating in updating drought communication plans (4) |
|  |  | \| 201\| Collaborating to distribute water and provide aid to vulnerable populations (4) |
|  |  | \| 71 \| Forming a collaborative team within the healthcare sector (5) |
|  |  | \| 80 \| Identifying key individuals in each sector for decision-making (25) |
| Education & Awareness | Healthcare staff training | \| 128\| Providing training for disaster response team members (4) |
|  |  | \| 129\| Educating healthcare providers on air quality health effects (2) |
|  |  | \| 130\| Educating local health centers on drought impacts (4) |
|  |  | \| 131\| Educating and training key partners (2) |
|  |  | \| 132\| Promoting educational initiatives for water health and nutrition (3) |
|  |  | \| 133\| Training healthcare workforce to recognize drought-related health conditions (5) |
|  |  | \| 134\| Training healthcare professionals in handling hazardous chemicals (5) |
|  |  | \| 135\| Training healthcare workers for health emergencies during high temperatures (5) |
|  |  | \| 136\| Training healthcare staff for power supply safety (5) |
|  |  | \| 137\| Raising awareness among healthcare workers about drought health effects (5) |
|  |  | \| 140\| Training healthcare staff to reduce water consumption (25) |
|  |  | \| 138\| Promoting awareness on utilizing care systems to track health outcomes (5) |
|  | Public awareness | \| 141\| Educating the public on water conservation during non-drought periods (4) |
|  |  | \| 142\| Issuing health guidelines for safe drinking water (2) |
|  |  | \| 143\| Raising awareness about water pollution risks (4) |
|  |  | \| 144\| Conducting public education in vulnerable communities (20) |
|  |  | \| 145\| Crafting messages for drought-related information dissemination (4) |
|  |  | \| 146\| Promoting sustainable practices for water use (3) |
|  |  | \| 147\| Delivering clear and appropriate information tailored to the audience (4) |
|  |  | \| 148\| Providing health education on food storage and handling (9) |
|  |  | \| 149\| Educating well owners on water purification measures (2) |
|  |  | \| 150\| Utilizing various communication channels for drought information (4) |
|  |  | \| 151\| Highlighting hand hygiene significance and alternatives (2) |
|  |  | \| 152\| Offering recommendations on cardiovascular health and lifestyle (20) |
|  |  | \| 153\| Consistently communicating the importance of well testing (2) |
|  |  | \| 154\| Providing essential drought-related information (4) |
|  |  | \| 155\| Issuing recommendations regarding air quality (2) |
|  |  | \| 156\| Awareness campaigns to reduce water consumption (25) |
|  |  | \| 157\| Shifting the tone of messages to collective cooperation (25) |
| Emergency Response & Crisis Management | Emergency plans | \| 47 \| Develop plans for relocating and deploying supplies during outbreaks (5) |
|  |  | \| 55 \| Create an emergency plan for the distribution of safe water (5) |
|  |  | \| 59 \| Arrange for patient evacuation to cooling stations (5) |
|  |  | \| 66 \| Activating mechanisms for continuity of essential services (3) |
|  |  | \| 70 \| Developing a local contingency plan for public health emergencies (11) |
|  |  | \| 76 \| Meeting the health needs of the affected population (3) |
|  |  | \| 77 \| Providing specialized healthcare services for early detection and disease management (3) |
|  |  | \| 78 \| Implementing mental and social healthcare support for the community (3) |
|  |  | \| 82 \| Providing sunscreen, hats, and ample drinking water for outdoor workers (5) |
|  | Disease and outbreak control | \| 90 \| Stockpiling laboratory kits for disease outbreak diagnosis (9) |
|  |  | \| 91 \| Offering sufficient sanitation facilities (9) |
|  |  | \| 92 \| Storing measles-containing vaccines alongside vitamin A supplements (9) |
|  |  | \| 102\| Maintaining communication with emergency units about waterborne diseases (11) |
|  |  | \| 106\| Developing a healthcare system for managing drought-related diseases (4, 25) |
| Innovation & Technology | Modern technologies | \| 35 \| Cultivating genetically modified crops resistant to heat and drought (32) |
|  |  | \| 45 \| Investing in green buildings with solar panels and water recycling (25) |
|  |  | \| 104\| Utilizing next-generation satellites for drought detection (18) |
|  |  | \| 183\| Identifying key partners and stakeholders (2) |
|  | Prediction and monitoring systems | \| 98 \| Establishing an early warning system for drought risk assessment (6, 13-15) |
|  |  | \| 101\| Developing an agricultural drought monitoring system (16, 17) |
|  |  | \| 113\| Utilizing advanced data to predict famine and food insecurity (13) |
| Economic Support & Policy | Financial assistance | \| 34 \| Offering subsidies and guaranteed income during drought seasons to reduce economic and psychological stress on farmers (22) |
|  |  | \| 115\| Providing seed capital for entrepreneurial initiatives (26) |
|  |  | \| 116\| Providing subsidies for fertilizers and agricultural inputs (26) |
|  |  | \| 202\| Greenhouse Gas Reduction (22) |
|  |  | \| 205\| Approval of support programs for agricultural projects (27) |
|  |  | \| 206\| Approval of loan interest cancellation program in vulnerable communities (35) |
|  | Drought mitigation policies | \| 97 \| Technological innovations, policy reforms, and regional cooperation (6) |
|  |  | \| 203\| Expediting issuance of new well permits (35) |
|  |  | \| 204\| Fire Containment Planning (30, 36) |

1. Mehdipour S, Nakhaee N, Khankeh H, Haghdoost AA. Impacts of drought on health: a qualitative case study from Iran. Int J Disaster Risk Reduct. 2022;76:103007. doi:10.1016/j.ijdrr.2022.103007.
2. National Center for Environmental Health (U.S.). When every drop counts: protecting public health during drought conditions—a guide for public health professionals. Atlanta (GA): Centers for Disease Control and Prevention; 2010. Accessed 2026 Aug 9. Available from: <https://stacks.cdc.gov/view/cdc/5758>.
3. Sena A, Barcellos C, Freitas C, Corvalan C. Managing the health impacts of drought in Brazil. Int J Environ Res Public Health. 2014;11(10):10737–51. doi:10.3390/ijerph111010737.
4. Centers for Disease Control and Prevention. Preparing for the health effects of drought: a resource guide for public health professionals. Atlanta (GA): CDC; 2018. Accessed 2026 Aug 9. Available from: <https://stacks.cdc.gov/view/cdc/61709>.
5. World Health Organization. Checklists to assess vulnerabilities in health care facilities in the context of climate change. Geneva (CH): World Health Organization; 2021. Accessed 2026 Aug 9. Available from: <https://www.who.int/publications/i/item/9789240022904>.
6. Mani ZA, Khorram-Manesh A, Goniewicz K. Global health emergencies of extreme drought events: historical impacts and future preparedness. Atmosphere. 2024;15(9):1137. doi:10.3390/atmos15091137.
7. Petkova EP, Celovsky AS, Tsai WY, Eisenman DP. Mental health impacts of droughts: lessons for the U.S. from Australia. In: Leal Filho W, Keenan JM, editors. Climate change adaptation in North America: fostering resilience and the regional capacity to adapt. Cham (CH): Springer; 2017. p. 289–304. doi:10.1007/978-3-319-53742-9_18.
8. Wijesekara NWANY, Wedamulla A, Perera S, Pesigan A, Ofrin RH. Assessment of drought resilience of hospitals in Sri Lanka: a cross-sectional survey. WHO South East Asia J Public Health. 2020;9(1):66–72. doi:10.4103/2224-3151.283000.
9. World Health Organization. Public health risk assessment and interventions: the Horn of Africa: drought and famine crisis. Geneva (CH): World Health Organization; 2011. Report No.: WHO/HSE/GAR/DCE/2011.3. Accessed 2026 Aug 9. Available from: <https://iris.who.int/bitstream/handle/10665/70691/WHO_HSE_GAR_DCE_2011.3_eng.pdf>.
10. Yusa A, Berry P, Cheng JJ, Ogden NH, Bonsal BR, Stewart RE, et al. Climate change, drought and human health in Canada. Int J Environ Res Public Health. 2015;12(7):8359–412. doi:10.3390/ijerph120708359.
11. Grigoletto JC, Cabral AR, Bonfim CV, Rohlfs DB, Silva EL, Queiroz FB, et al. Management of health sector actions in drought situations [Gestão das ações do setor saúde em situações de seca e estiagem]. Cien Saude Colet. 2016;21(3):709–18. doi:10.1590/1413-81232015213.26212015.
12. Salvador C, Nieto R, Vicente-Serrano SM, García-Herrera R, Gimeno L, Vicedo-Cabrera AM. Public health implications of drought in a climate change context: a critical review. Annu Rev Public Health. 2023;44:213–32. doi:10.1146/annurev-publhealth-071421-051636.
13. START Center, Sankar-Gorton L, Lubeck-Schricker M, Hossain R, Iwu CD, Hess J, et al. The impact of climate change on public health in humanitarian settings [version 1; not peer reviewed]. Gates Open Res. 2024;8:71. doi:10.21955/gatesopenres.1117114.1.
14. Sari PN, Djafri D, Gusti A. Climate change: how is the adaptation of public health services? Case studies in the city of Padang. J Phys Conf Ser. 2019;1351(1):012108. doi:10.1088/1742-6596/1351/1/012108.
15. Lombe P, Carvalho E, Rosa-Santos P. Drought dynamics in sub-Saharan Africa: impacts and adaptation strategies. Sustainability. 2024;16(22):9902. doi:10.3390/su16229902.
16. Andriesse M. Drought is insurable [Internet]. Swiss Re. Zurich (CH): Swiss Re; 2021 Jun 16 [cited 2026 Aug 9]. Available from: <https://www.swissre.com/risk-knowledge/mitigating-climate-risk/drought-is-insurable.html>.
17. Enenkel M, See L, Bonifacio R, Boken V, Chaney N, Vinck P, et al. Drought and food security—improving decision-support via new technologies and innovative collaboration. Glob Food Sec. 2015;4:51–5. doi:10.1016/j.gfs.2014.08.005.
18. Kogan F, Guo W, Yang W. Drought and food security prediction from NOAA new generation of operational satellites. Geomat Nat Hazards Risk. 2019;10(1):651–66. doi:10.1080/19475705.2018.1541257.
19. Abunyewah M, Byrne MK, Keane CA, Bressington D. Developing psychological resilience to the impact of drought. Int J Environ Res Public Health. 2023;20(4):3465. doi:10.3390/ijerph20043465.
20. Hart CR, Berry HL, Tonna AM. Improving the mental health of rural New South Wales communities facing drought and other adversities. Aust J Rural Health. 2011;19(5):231–8. doi:10.1111/j.1440-1584.2011.01225.x.
21. Missouri Department of Mental Health; University of Missouri Extension. Tips for coping with drought-related stress [Internet]. Jefferson City (MO): Missouri Department of Mental Health; 2012 [cited 2026 Aug 9]. Available from: <https://dmh.mo.gov/media/pdf/tips-coping-drought-related-stress>.
22. Padhy SK, Sarkar S, Panigrahi M, Paul S. Mental health effects of climate change. Indian J Occup Environ Med. 2015;19(1):3–7. doi:10.4103/0019-5278.156997.
23. Hernández-López JA, Puerta-Cortés DX, Andrade HJ. Predictive analysis of adaptation to drought of farmers in the central zone of Colombia. Sustainability. 2024;16(16):7210. doi:10.3390/su16167210.
24. Orievulu KS, Iwuji CC. Institutional responses to drought in a high HIV prevalence setting in rural South Africa. Int J Environ Res Public Health. 2021;19(1):434. doi:10.3390/ijerph19010434.
25. Quintana AV, Mayhew SH, Kovats S, Gilson L. Resilient health systems in action: how actor relationships and organizational adaptation shaped the health sector's response to the 2015–2018 Western Cape drought. Oxf Open Clim Chang. 2025;5(1):kgaf014. doi:10.1093/oxfclm/kgaf014.
26. Rosen JG, Mulenga D, Phiri L, Okpara N, Brander C, Chelwa N, et al. "Burnt by the scorching sun": climate-induced livelihood transformations, reproductive health, and fertility trajectories in drought-affected communities of Zambia. BMC Public Health. 2021;21(1):1501. doi:10.1186/s12889-021-11560-8.
27. International Federation of Red Cross and Red Crescent Societies; Save the Children. Public awareness and public education for disaster risk reduction: action-oriented key messages for households and schools. 2nd ed. Geneva (CH): IFRC; 2018. Accessed 2026 Aug 9. Available from: <https://www.ifrc.org/sites/default/files/PAPE-2.0-English.pdf>.
28. Elstow L, Rojas Parra F, MacAskill K. Tracking climate adaptation in hospitals: an inventory of structural measures. Clim Risk Manag. 2024;46:100657. doi:10.1016/j.crm.2024.100657.
29. Makvandi M, Li W, Ou X, Chai H, Khodabakhshi Z, Fu J, et al. Urban heat mitigation towards climate change adaptation: an eco-sustainable design strategy to improve environmental performance under rapid urbanization. Atmosphere. 2023;14(4):638. doi:10.3390/atmos14040638.
30. Centers for Disease Control and Prevention. Drought communication toolkit [Internet]. Drought and Health. Atlanta (GA): CDC; 2024 Apr 15 [cited 2026 Aug 9]. Available from: <https://www.cdc.gov/drought-health/toolkit/index.html>.
31. United States Environmental Protection Agency. Drought resilience and water conservation [Internet]. US EPA. Washington (DC): EPA; 2026 Mar 18 [cited 2026 Aug 9]. Available from: <https://www.epa.gov/water-research/drought-resilience-and-water-conservation>.
32. Patz JA, Hatch MJ. Public health and global climate disruption. Public Health Rev. 2013;35(1):12. doi:10.1007/BF03391697.
33. World Health Organization. Operational framework for building climate resilient and low carbon health systems. Geneva (CH): World Health Organization; 2023. Accessed 2026 Aug 9. Available from: <https://www.who.int/publications/i/item/9789240081888>.
34. Sartore GM, Kelly B, Stain HJ. Drought and its effect on mental health—how GPs can help. Aust Fam Physician. 2007;36(12):990–3.
35. Jedd T, Fragaszy SR, Knutson C, Hayes MJ, Fraj MB, Wall N, et al. Drought management norms: is the Middle East and North Africa region managing risks or crises? J Environ Dev. 2021;30(1):3–40. doi:10.1177/1070496520960204.
36. Smith LT, Aragão LEOC, Sabel CE, Nakaya T. Drought impacts on children's respiratory health in the Brazilian Amazon. Sci Rep. 2014;4:3726. doi:10.1038/srep03726.
